# Human Leucocyte Antigen-G (HLA-G) and Its Murine Functional Homolog Qa2 in the *Trypanosoma cruzi* Infection

**DOI:** 10.1155/2015/595829

**Published:** 2015-01-20

**Authors:** Fabrício C. Dias, Celso T. Mendes-Junior, Maria C. Silva, Fabrine S. M. Tristão, Renata Dellalibera-Joviliano, Philippe Moreau, Edson G. Soares, Jean G. Menezes, André Schmidt, Roberto O. Dantas, José A. Marin-Neto, João S. Silva, Eduardo A. Donadi

**Affiliations:** ^1^Departamento de Clínica Médica, Faculdade de Medicina de Ribeirão Preto, Universidade de São Paulo, Avenida Bandeirantes 3900, 14049-900 Ribeirão Preto, SP, Brazil; ^2^Departamento de Química, Faculdade de Filosofia, Ciências e Letras de Ribeirão Preto, Universidade de São Paulo, Avenida Bandeirantes 3900, 14049-900 Ribeirão Preto, SP, Brazil; ^3^Departamento de Bioquímica e Imunologia, Faculdade de Medicina de Ribeirão Preto, Universidade de São Paulo, Avenida Bandeirantes 3900, 14049-900 Ribeirão Preto, SP, Brazil; ^4^Departamento de Morfologia, Fisiologia e Patologia Básica, Faculdade de Odontologia de Ribeirão Preto, Universidade de São Paulo, Avenida Bandeirantes 3900, 14049-900 Ribeirão Preto, SP, Brazil; ^5^Departamento de Cirurgia e Anatomia, Faculdade de Medicina de Ribeirão Preto, Universidade de São Paulo, Avenida Bandeirantes 3900, 14049-900 Ribeirão Preto, SP, Brazil; ^6^CEA, Institute of Emerging Diseases and Innovative Therapies, Research Division in Hematology and Immunology, Saint-Louis Hospital, 1 Avenue Claude Vellefaux, 75475 Paris, France; ^7^Departamento de Patologia e Medicina Legal, Faculdade de Medicina de Ribeirão Preto, Universidade de São Paulo, Avenida Bandeirantes 3900, 14049-900 Ribeirão Preto, SP, Brazil

## Abstract

Genetic susceptibility factors, parasite strain, and an adequate modulation of the immune system seem to be crucial for disease progression after *Trypanosoma cruzi* infection. HLA-G and its murine functional homolog Qa2 have well-recognized immunomodulatory properties. We evaluated the *HLA-G* 3′ untranslated region (3′UTR) polymorphic sites (associated with mRNA stability and target for microRNA binding) and HLA-G tissue expression (heart, colon, and esophagus) in patients presenting Chagas disease, stratified according to the major clinical variants. Further, we investigated the transcriptional levels of Qa2 and other pro- and anti-inflammatory genes in affected mouse tissues during *T. cruzi* experimental acute and early chronic infection induced by the CL strain. Chagas disease patients exhibited differential *HLA-G* 3′UTR susceptibility allele/genotype/haplotype patterns, according to the major clinical variant (digestive/cardiac/mixed/indeterminate). HLA-G constitutive expression on cardiac muscle and colonic cells was decreased in Chagasic tissues; however, no difference was observed for Chagasic and non-Chagasic esophagus tissues. The transcriptional levels of *Qa2* and other anti and proinflammatory (*CTLA-4, PDCD1, IL-10, INF-γ*, and *NOS-2*) genes were induced only during the acute *T. cruzi* infection in BALB/c and C57BL/6 mice. We present several lines of evidence indicating the role of immunomodulatory genes and molecules in human and experimental *T. cruzi* infection.

## 1. Introduction

Chagas disease or American trypanosomiasis is caused by the parasite* Trypanosoma cruzi*, which may produce acute and chronic manifestations. During the acute phase, most cases are indeterminate, but some patients may develop severe myocarditis or meningoencephalitis, which can be lethal. The chronic phase exhibits four major clinical variants: (i) the indeterminate form represents 50–70% of cases and develops without evident clinical and pathological signs [1]; (ii) the cardiac form encompasses 10–20% of cases and usually presents progressive congestive heart failure, various cardiac arrhythmias, thromboembolic events, and sudden death [1, 2]; (iii) the digestive form (8–10% of cases) is characterized by clinical signs of megaesophagus, megacolon, or both; (iv) the cardiodigestive or mixed form (8% of cases) comprises clinical and pathological signs of cardiac and digestive involvement. These clinical syndromes are considered to be a consequence of autonomic neuronal loss, microvascular derangements, and chronic organ inflammation directly dependent on parasite persistence or by immune system cells [3, 4].

In parallel with the chronic parasite infection, the immune response against parasite antigens is associated with the Chagas heart disease pathogenesis. Key features of the immunity in the Chagas disease include (i) predominance of partially activated T CD8 lymphocytes in cardiac inflammatory infiltrates [5], accompanied by high production of nitric oxide (NO), IL-12, monocyte chemoattractant protein-1 (MCP-1), and IFN-*γ* by infiltrating macrophages; (ii) preponderant IFN-*γ* production by cytolytic natural killer (NK) cells in the acute phase of the disease to control tissue and systemic parasite burden; and (iii) polyclonal B lymphocyte response [6–13].

Cytokine patterns in Chagas disease are characterized by T-helper (Th)1 polarization in the acute phase, with predominance of IFN-*γ* and TNF production. During the chronic phase, a coexistence of the Th1 (IL-12, IFN-*γ*, and TNF) and Th2 profiles (IL-4 and IL-10) may be observed, and the equilibrium between these profiles may be relevant to disease morbidity [3, 14, 15]; that is, a Th1 response aggravates and a Th2 response produces a better outcome in murine trypanosomiasis and human Chagas disease [16–20]. Besides cytokines, other immunomodulatory molecules such as PD-1 and CTLA-4 are also involved in acute and chronic trypanosomiasis [20–25]. In this context, immunomodulatory molecules may play an important role for disease evolution, controlling or exacerbating the immune response against* T. cruzi* itself or tissue modifications induced by parasites.

Among immunomodulatory molecules, the human leucocyte antigen- (HLA-) G is a nonclassical histocompatibility class Ib molecule, which has a well-recognized role in controlling several branches of the immune response, inhibiting T cell proliferation, NK, and cytotoxic T lymphocytes, inducing regulatory T cells and tolerizing dendritic cells [26]. These effects are primarily due to the preferential interaction of the HLA-G molecule with the Ig-like transcript (ILT)2, ILT4, and killer cell immunoglobulin-like (KIR2DL4) receptors, which induce inhibitory intracellular signals via tyrosine-based (ITIM) motifs [27].

*HLA-G* mRNA has restricted expression in nonpathological conditions, for organs such as the placenta, thymus, heart, intestines, brain, and skin [28], and the constitutive expression of the HLA-G molecule has been observed on placenta, thymus, cornea, pancreas, brain, erythroid cells, and blood cell surfaces [29–34]. On the other hand, HLA-G neoexpression has been observed in several situations, including tumors, viral infections, engrafted tissues, and autoimmune and inflammatory diseases. Depending on the underlying condition, the expression may be advantageous when the blockade of the immune response is desirable, such as in autoimmune disorders and in allografting, whereas HLA-G expression in tumors and chronic viral infections may be harmful [35, 36].

At the coding region, the* HLA-G* gene presents limited polymorphisms compared to classical HLA class I (*HLA-A/B/C*) genes, exhibiting only 50 alleles (The International Immunogenetics Database (IMGT), v3.16.0) and coding 16 different membrane-bound full length proteins. The regulatory regions also present several polymorphisms that coincide with or are close to binding sites for transcription factors (promoter region) or are targets for microRNA binding (3′ untranslated region (3′UTR)). At least 29 variation sites have been described at the promoter region [37] and 16 variants have been identified at the* HLA-G* 3′UTR [38]. Considering that the regulatory regions are involved on the magnitude of* HLA-G* gene expression, the study of these regions is relevant. In the healthy Brazilian population, at least eight* HLA-G* 3′UTR variation sites have been described [37, 39], and some of them have been associated with plasma soluble HLA-G (sHLA-G) levels [40].

The HLA-G murine functional homolog Qa2, encoded by* H2-Q7/Q9* gene, also presents restricted tissue expression, and Qa2 mRNA has been reported in thymus, liver, intestines, spleen, placenta, and brain. Qa2 also modulates the immune response by interacting with as yet unidentified NK cell receptors [41–45].

Little information is available regarding HLA-G and the Qa2 murine functional homolog in acute and chronic parasitic diseases. Considering that (i) no information is available regarding the role of these immunoregulatory molecules in* T. cruzi* infection; (ii) Chagas disease is a chronic disorder, in which several mechanisms of immunomodulation have been described in association with its pathogenesis; (iii) clinical variants of Chagas disease may depend on parasite and host genetic factors; (iv) experimental trypanosomiasis may help in the understanding of the human disease counterpart, we designed a study encompassing the HLA-G tissue expression and* HLA-G* 3′UTR polymorphic site typing in patients presenting Chagas disease, stratified according to major clinical variants. In addition, we evaluated the transcriptional level of the* H2-Q7/Q9* (Qa2), and other immunoregulatory* H2-T23* (Qa1),* CTLA-4,* and* PDCD1* (PD-1) genes in mouse (BALB/c and C57BL/6) affected tissues during experimental acute and early chronic infection caused by the CL strain of* T. cruzi* and correlated the expression of these genes with other mediators of inflammation, including* INF-γ*, inducible nitric oxide synthase (*NOS-2*), and* IL-10*.

## 2. Methods

### 2.1. Subjects

The protocol of the study was approved by the local Research Ethics Committee (Protocol number 11237/2009) and written informed consent was obtained from all participants. A total of 177 chronic Chagas disease patients exhibiting positive serology for* T. cruzi* antigens followed at the Divisions of Cardiology and Gastroenterology of the Department of Medicine of the School of Medicine of Ribeirão Preto, University of São Paulo, Brazil, and 155 healthy individuals from the same region of patients, exhibiting negative serology for* T. cruzi* infection, were studied. The Chagas disease patients were submitted to clinical and laboratory examination. Electro- and echocardiography, esophagus and colon contrast X-ray examinations, and esophagus electromanometry were performed to classify patients into cardiac (*n* = 52, exhibiting or not heart failure), digestive (*n* = 62, exhibiting megaesophagus, megacolon, or both), cardiodigestive or mixed (*n* = 24, presenting any combination of cardiac and digestive forms), and indeterminate (*n* = 39) variants.

### 2.2. DNA Extraction and Genotyping

Genomic DNA was extracted from peripheral blood leucocytes using a standard salting-out procedure. Genotyping of the variation sites at* HLA-G* 3′UTR was performed by sequencing analyses as previously described [39].

### 2.3. Immunohistochemical Analysis of HLA-G

Fifty-four specimens of tissues obtained from Chagas disease deceased patients were analyzed, of which 30 were derived from heart, 13 from colon, and 11 from esophagus. In parallel, 20 tissue specimens (8 from heart, 6 from esophagus, and 6 from sigmoid colon) of deceased individuals without Chagas disease and exhibiting no histopathological tissue alteration were analyzed. Three specimens of trophoblast tissue (positive control for HLA-G expression) were also analyzed. The immunohistochemical analysis was performed using the primary antibody MEM/G9 (HLA-G1 and sHLA-G5 specific mouse IgG) (Exbio, Prague, Czech Republic), diluted 1 : 200, as previously described [46]. Briefly, after defining the range of brown color considered being positive, images were converted to 256 shades (8-bit) of gray. Then, the grayscale images were converted into a binary (black and white) variable to define the cutoff point. The threshold was adjusted, and the brown areas became black portions in the binary image [47]. Immunostained areas were evaluated using the ImageJ software (National Institutes of Health, Bethesda, MD).

### 2.4. Experimental Infections and Transcriptional Level Analysis

Male eight-week-old C57BL/6 and BALB/c mice were intraperitoneously (IP) injected with bloodstream CL strain forms of* T. cruzi*, using three infected and three noninfected animals in two independent experiments. The mice were cared for according to the institutional guidelines on ethics in animal experiments and all protocols were approved by the local Ethics Committee on Animal Care and Research (process number 172/2009).

For the acute infection, 10^3^ bloodstream forms were IP injected. Mouse survival was verified daily for 30 days and the parasitemia was quantified microscopically by counting the parasites in 5 *μ*L of citrated blood obtained from the tail lateral vein from day 7 until day 29 after infection. Parasite load was analyzed in the heart at day 24 after infection [48]. For the early chronic infection, 10^2^ bloodstream forms of* T. cruzi* were IP injected and mouse survival was verified daily for 60 days. Parasite load was analyzed in the heart at day 60 after infection.

Gene expression was detected in the heart and esophagus at 24 and 60 days after infection (dpi), for acute and early chronic infections, respectively. Although Qa2, Qa1, and PD-1 are the encoded molecules of the* H2-Q7/Q9*,* H2-T23,* and* PDCD1* genes, respectively, to facilitate understanding we will refer in this text to* Qa2, Qa1,* and* PD-1* expression as synonyms of their respective gene expressions.

Total RNA was extracted from tissues using the TRIzol reagent (Invitrogen, Carlsbad, CA) and was reverse transcribed to obtain cDNA with the SuperScript III (Invitrogen) transcriptase reverse. SYBR Green Mix-based real-time quantitative PCR assays were performed using a StepOnePlus system (Applied Biosystems, Foster City, CA). Data were normalized according to the expression of the glyceraldehyde phosphate dehydrogenase (*GADPH*) housekeeping gene and relative expression of each mRNA was calculated with the 2^−∆∆Ct^ method [49]. Primer sequences included (i)* H2-Q7/Q9*-forward 5′-ATG GCG ACC ATT GCT GTT GT-3′,* H2-Q7/Q9*-reverse 5′-TCC AAT GAT GGC CAC AGC T-3′; (ii)* H2-T23*-fwd 5′-GCA CAA GTC AGA GGC AGT CG-3′,* H2-T23*-rev 5′-TGC AGG TAT GCC CTC TGT TG-3′; (iii)* CTLA-4*-fwd 5′-ACC TCT GCA AGG TGG AAC TCA-3′,* CTLA-4*-rev 5′-CCA TGC CCA CAA AGT ATG GC-3′; (iv)* PD-1*-fwd 5′-TTC AGG TTT ACC ACA AGC TGG-3′,* PD-1*-rev 5′-TGA CAA TAG GAA ACC GGG AA-3′; (v)* INF-γ*-fwd 5′-GCA TCT TGG CTT TGC AGC T-3′,* INF-γ*-rev 5′-CCT TTT TCG CCT TGC TGT TG-3′; (vi)* NOS-2*-fwd 5′-CGA AAC GCT TCA CTT CCA A-3′,* NOS-2*-rev 5′-TGA GCC TAT ATT GCT GTG GCT-3′; (vii)* IL-10*-fwd 5′-TGG ACA ACA TAC TGC TAA CC-3′,* IL-10*-rev 5′-GGA TCA TTT CCG ATA AGG CT-3′; (viii)* GAPDH*-fwd 5′-TGC AGT GGC AAA GTG GAG AT-3′,* GADPH*-rev 5′-CGT GAG TGG AGT CAT ACT GGA A-3′.

### 2.5. Statistical Analysis

Allele and genotype frequencies were estimated by direct counting, and adherences of phenotypical proportions to expectations under the Hardy-Weinberg equilibrium (HWE) were tested by the complete enumeration method using the GENEPOP 3.4 software [50]. Linkage disequilibrium (LD) between variation sites at* HLA-G* 3′UTR was evaluated by means of a likelihood ratio test of linkage disequilibrium implemented at the ARLEQUIN software [51].* HLA-G* 3′UTR haplotypes of each individual were inferred for each group of patients (stratified according to the four clinical variants) and for the whole group of patients. Two distinct computational methods that do not take any prior information into account were used for this purpose: (1) the EM algorithm [52] implemented with the PL-EM software [53] and (2) a coalescence-based method implemented in the PHASE v2 software [54]. Therefore, the haplotype pair of each subject was independently inferred by four approaches; whenever these four approaches resulted in nonconsensual inference for a given subject, he was removed from all procedures that used haplotype data as input. The frequency of each allele, genotype, or haplotype was compared between patients and controls by the two-sided Fisher exact test, with the aid of the GraphPad Instat 3.05 software, which was also used to estimate the Odds Ratio (OR) and its 95% Confidence Interval (CI).

For the HLA-G tissue expression, gene expression, and parasitism load, statistical analyses were performed using Student's *t*-test or Mann-Whitney tests and the relationship between immunomodulatory and mediators of inflammation genes was performed using the Spearman Correlation, both with the aid of the GraphPad Prism 5 v5.0b software. To facilitate data presentation, variables were expressed as mean ± SEM.

For all analyses, significance was defined at *P* < 0.05.

## 3. Results

### 3.1. Genotype and Allele Frequencies of Variation Sites at* HLA-G* 3′UTR

*HLA-G* 3′UTR variation sites were compared between (i) patients (considered as whole) with controls (healthy individuals); (ii) patients presenting clinically detectable disease (CDM) (cardiac (C) plus digestive (D) plus mixed (M) forms) with controls; (iii) patients presenting CDM with indeterminate patients (indeterminate form—I); and (iv) patients stratified according to the four clinical variants with each other and with controls. Association tests were performed for alleles and genotypes of each variation site.

The following* HLA-G* 3′UTR variation sites were observed in this study: 14-base pair insertion/deletion (I/D; rs66554220); +3001C/T (rs not available); +3003T/C (rs115689421); +3010C/G (rs116152775); +3027A/C (rs115810666); +3035C/T (rs115100128); +3142G/C (rs115928989); +3187A/G (rs114317070); and +3196C/G (rs115045214). Overall, these variation sites adhered to the Hardy-Weinberg Equilibrium, except +3010C/G in cardiac and whole group of patients and +3142G/C in digestive and whole group of patients.  Table 1 shows the genotype and allele frequencies in patients with chronic Chagas disease and controls.

14-base pair I/D and +3001C/T: no significant differences were observed when the five types of comparisons were performed. The +3001T allele was observed in only one Chagas disease patient presenting exclusively the digestive form.

+3003T/C: the +3003CC genotype was not observed in Chagasic patients. Compared to controls, (i) the +3003TC genotype frequency was decreased in the whole group of patients (*P* = 0.0131) and in the digestive form (*P* = 0.0462); (ii) the frequency of the +3003C allele was decreased in the whole group of patients (*P* = 0.0101) and in patients exhibiting the digestive form (*P* = 0.0448); (iii) +3003TT genotype frequency was overrepresented in the whole group (*P* = 0.0091) and in digestive (*P* = 0.0321) patients; and (iv) +3003T allele frequency was increased in whole group (*P* = 0.0101) and in the digestive group (*P* = 0.0448) of patients.

+3010C/G: compared to controls, the +3010CG genotype was underrepresented in the whole group of patients (*P* = 0.0322) and in those presenting the cardiac variant (*P* = 0.0098).

+3027A/C: a decreased frequency of the +3027AC genotype was observed in the digestive form compared to control (*P* = 0.0069) and to patients with the indeterminate (*P* = 0.0026), cardiac (*P* = 0.0076), and mixed (*P* = 0.0050) forms. The frequency of the +3027CC genotype was increased in patients exhibiting the digestive form compared to indeterminate (*P* = 0.0026), cardiac (*P* = 0.0076), mixed (*P* = 0.0050), and control (*P* = 0.0071) groups. The +3027AA and +3027AC genotypes were not observed in patients with the digestive form. As consequence, the digestive group exhibited a decreased frequency of the +3027A allele compared to controls (*P* = 0.0046) and to patients with the indeterminate (*P* = 0.0029), cardiac (*P* = 0.0083), and mixed (*P* = 0.0055) forms. The +3027C allele presented the opposite association pattern.

+3035C/T: the +3035CC genotype was overrepresented in patients with the digestive form compared to control (*P* = 0.0024) and to patients with cardiac (*P* = 0.0247) and mixed (*P* = 0.0404) forms. A decreased frequency of the +3035CT genotype was observed in patients exhibiting the digestive form compared to mixed (*P* = 0.0404) and control (*P* = 0.0095) groups. The +3035C allele was overrepresented in the group of patients with the digestive form compared to control (*P* = 0.0020), indeterminate (*P* = 0.0346), cardiac (*P* = 0.0118), and mixed (*P* = 0.0489) groups. The +3035T allele presented the opposite association pattern.

+3142G/C: the +3142GG genotype frequency was significantly lower in digestive patients compared to indeterminate (*P* = 0.0449), cardiac (*P* = 0.0363), and mixed (*P* = 0.0346) forms. The +3142GC genotype frequency was lower in cardiac patients compared to digestive (*P* = 0.0211) and control (*P* = 0.0061) groups. In addition, +3142GC genotype frequency was decreased in the whole group of patients group compared to the control group (*P* = 0.0459).

+3187A/G: compared to controls, an increased frequency of the +3187GG genotype was observed in the whole group (*P* = 0.0169) and in the group of patients with the cardiac form (*P* = 0.0459).

+3196C/G: patients exhibiting the mixed form presented a decreased frequency of the +3196CC genotype (*P* = 0.0499) and an increased frequency of +3196GC genotype (*P* = 0.0258). In addition, +3196CG genotype was increased in the mixed group compared to the indeterminate variant (*P* = 0.0188).

When CDM patients (excluding the indeterminate group) were compared to controls, the following results were observed: (i) the +3003CT genotype frequency was decreased in patients (*P* = 0.0128); (ii) the frequency of the +3003TT genotype was increased in patients (*P* = 0.0088); (iii) +3003C allele frequency was decreased in patients (*P* = 0.0097) and +3003T allele presented the opposite association pattern (*P* = 0.0097); (iv) the +3187GG genotype frequency was overrepresented in patients (*P* = 0.0282); (v) +3196CC genotype frequency was decreased (*P* = 0.0259) and +3196GC genotype frequency was increased (*P* = 0.0325) in patients.

When CDM patients were compared to the indeterminate form, only the +3196GC genotype was increased in CDM patients (*P* = 0.0440). On the other hand, when indeterminate patients were compared to healthy controls, no significant results were observed.

Table 2 shows the Odds Ratio and 95% Confidence Interval values obtained for all significant comparisons.

### 3.2. Haplotype Frequency of Variation Sites at* HLA-G* 3′UTR

To further understand how the ensemble of variation sites participate in Chagas disease susceptibility and considering that these variation sites were in linkage disequilibrium (results not shown), we analyzed the frequency of the* HLA-G* 3′UTR haplotypes observed for patients and controls, and the results of the statistical analyses are shown in Tables 3(a) and 3(b). Eleven out of 177 patients have been removed from all analyses that used haplotype data as input, since results for the four approaches of haplotype inference resulted in nonconsensual results. The names of the 3′UTR haplotypes (UTR-1, UTR-2, etc.) follow the nomenclature previously described by our group [39], which has been extensively used in the literature.

Compared to controls, (i) UTR-4 was underrepresented in the whole group of patients (*P* = 0.0005), CDM (*P* = 0.0008), digestive (*P* = 0.0274), and cardiac (*P* = 0.0043) forms; (ii) UTR-13 was overrepresented in the indeterminate group (*P* = 0.0320); (iii) UTR-14 was overrepresented in cardiac patients (*P* = 0.0135). A decreased frequency of the UTR-7 haplotype was observed in the digestive group compared to indeterminate (*P* = 0.0056), mixed (*P* = 0.0053), and control (*P* = 0.0048) groups. In addition, UTR-13 was also increased in patients presenting the indeterminate form when compared to CDM patients (*P* = 0.0415).

### 3.3. HLA-G Expression in Specimens Obtained from Chronic Chagas Disease Patients

**Figure 1:**
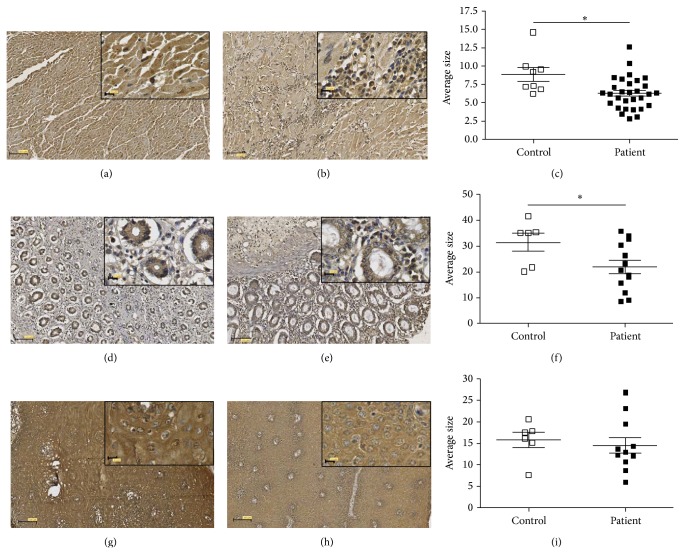
Immunohistochemical staining for HLA-G. Representative photomicrographs ((a), (b), (d), (e), (g), and (h)) and average size ((c), (f), and (i)) of HLA-G-stained cells in heart ((a)–(c)), colon ((d)–(f)), and esophagus ((g)–(i)) of controls ((a), (d), and (g)) and patients ((b), (e), and (h)). The insert boxes show a higher magnification of the HLA-G-stained cells in each tissue. Scale bar 100 *μ*m, 12 *μ*m (insert). ((c), (f), and (i)) Data are expressed as mean ± SEM. ^*^ *P* < 0.05 indicating statistical significance compared with controls.

HLA-G molecule expression was evaluated in the major organs affected by the disease, including heart, colon, and esophagus. Compared to non-Chagasic tissues, HLA-G expression was significantly decreased in Chagasic heart (*P* = 0.0105; Figures 1(a), 1(b), and 1(c)) and colon (*P* = 0.0485, Figures 1(d), 1(e), and 1(f)). HLA-G expression in individuals without Chagas disease was primarily observed on cardiac muscle cells and no cellular infiltration was observed (Figure 1(a)), whereas specimens from Chagas patients exhibiting cardiomegaly showed lesser HLA-G expression on cardiac muscle cells together with an infiltration of mononuclear cells (lymphocytes and plasma cells) exhibiting HLA-G expression (Figure 1(b)). The HLA-G immunolabeling in esophagus of Chagas patients with esophagomegaly was closely similar to that observed for individuals without Chagas disease (Figures 1(g), 1(h), and 1(i)).

### 3.4. *Qa2* Expression during Experimental Acute and Early Chronic Infections

The transcriptional level of* Qa2* in acute and early chronic infections was studied in heart and esophagus specimens obtained from BALB/c and C57BL/6 infected mice, using the* T. cruzi* CL strain.

**Figure 2:**
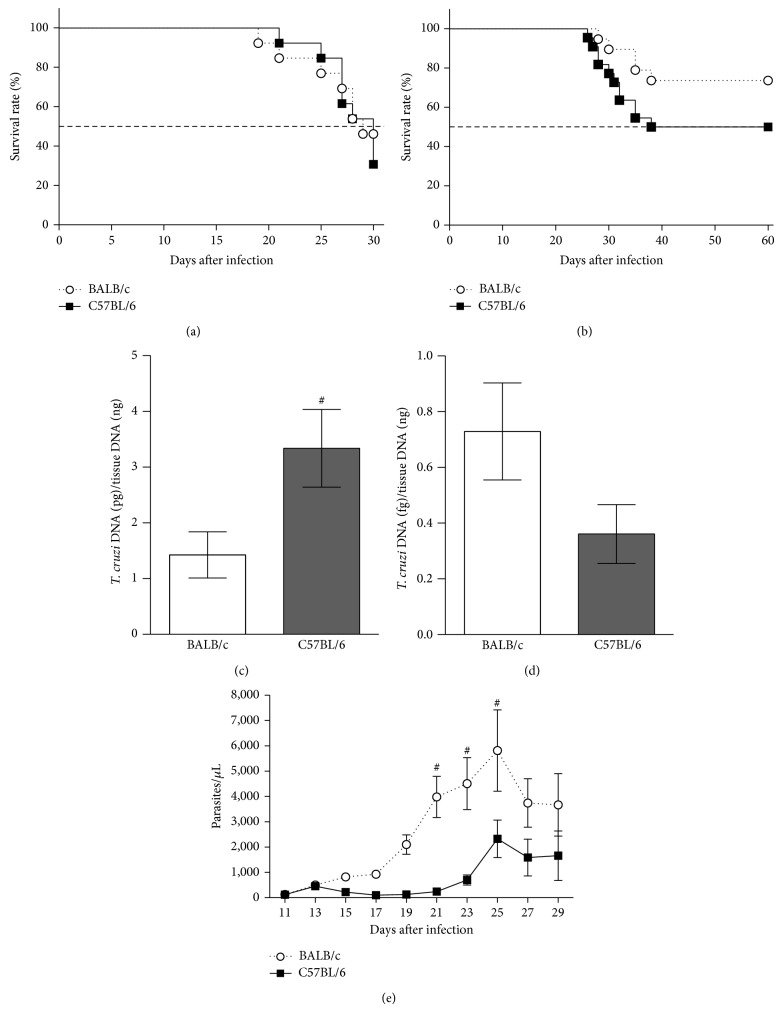
Survival, parasite load, and parasitism of* T. cruzi* infected mice. The male 8-week-old BALB/c and C57BL/6 mice were intraperitoneally infected with 10^3^ or 10^2^ bloodstream CL strain forms for acute and early chronic infections, respectively. For acute infection, the survival was observed daily during 35 days (a) and for early chronic infection the survival was observed daily during 60 days (b). The parasite load was determined in the heart at day 24 after infection for acute infection (c) and at day 60 after infection for early chronic infection (d). Parasitemia was quantified microscopically for 29 days (e). The data represent the mean ± SEM (*n* = 6 mice/group). ^#^ *P* < 0.05, comparison between BALB/c and C57BL/6 infected mice.

First, we characterized the acute and early chronic infections regarding the animal survival rate, tissue parasite load, and blood parasitism. Survival rates showed no significant differences for BALB/c and C57BL/6 mice for both acute (Figure 2(a)) and early chronic (Figure 2(b)) infections. During acute infection, the heart parasite load was 2-fold increased in C57BL/6 mice in relation to BALB/c group (*P* < 0.05; Figure 2(c)). No significant difference was observed between the two groups during the early chronic infection (Figure 2(d)). In addition, when we analyzed the bloodstream forms in the acute infection, BALB/c mice showed an increased parasitism level at days 21, 23, and 25 compared to C57BL/6 mice (*P* < 0.05; Figure 2(e)).

**Figure 3:**
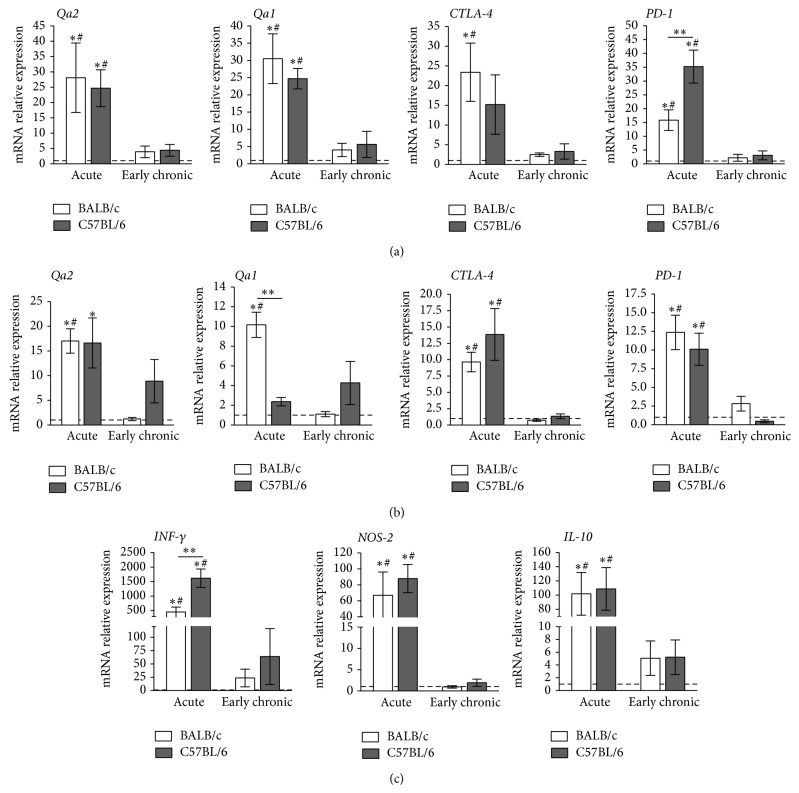
Transcriptional level in the heart and esophagus of* T. cruzi* infected mice. Transcript levels for immunomodulatory genes were measured by qRT-PCR in whole heart (a) and esophagus (b) homogenates obtained from infected mice at days 24 and 60 after infection for acute and early chronic infections, respectively. Transcriptional levels for INF-*γ*, NOS-2, and IL-10 were measured by qRT-PCR in whole heart homogenate (c). Uninfected mice are represented by dashed lines. The results are expressed as relative expression. The data represent the mean ± SEM (*n* = 6 mice/group). ^*^ *P* < 0.05 indicating statistical significance compared with noninfected mice. ^#^ *P* < 0.05, comparison between acute and early chronic infections. ^**^ *P* < 0.05, comparison between BALB/c and C57BL/6 infected mice.

During acute infection, the heart and esophagus transcriptional levels of* Qa2* were significantly increased for both mouse strains, when compared to noninfected mice. Compared to the control group, the heart expression of* Qa2* in BALB/c and C57BL/6 was 28-fold and 25-fold increased, respectively (*P* < 0.05; Figure 3(a)), and the esophagus expression of* Qa2* was 17-fold and 16-fold increased in BALB/c and C57BL/6, respectively (*P* < 0.05; Figure 3(b)).

Acutely infected BALB/c mice showed increased transcriptional levels of* Qa2* in heart and esophagus, which were 7-fold and 14-fold higher than early chronically infected mice, respectively (*P* < 0.05; Figures 3(a) and 3(b)). The transcriptional level of* Qa2* was 6-fold higher in heart of acutely infected C57BL/6 mice compared to early chronic infection (*P* < 0.05; Figure 3(a)). No significant difference in terms of* Qa2* expression was observed between noninfected and early chronically infected mice in heart and esophagus of BALB/c and C57BL/6 mice (Figures 3(a) and 3(b)).

To verify whether other known immunomodulatory genes were concomitantly modulated during the infection by CL strain of* T. cruzi*, we analyzed the transcriptional levels of* Qa1*,* CTLA-4* and* PD-1* genes. Our results showed that all of them were significantly augmented in heart and esophagus of BALB/c strain when compared to noninfected and early chronically infected mice (*P* < 0.05; Figures 3(a) and 3(b)). C57BL/6 mice showed an increased transcriptional level of* Qa1* in heart and increased levels of* CTLA-4* in esophagus and* PD-1* in both heart and esophagus, during the acute infection (*P* < 0.05), compared to controls and early chronically infected animals (Figures 3(a) and 3(b)). Moreover, during the acute infection,* Qa1* was 4-fold higher in esophagus of BALB/c strain compared to C57BL/6 animals, and* PD-1* expression was 2-fold higher in heart of C57BL/6 mice compared to BALB/c strain (*P* < 0.05; Figures 3(a) and 3(b)). No significant difference was observed between noninfected and early chronically infected mice in heart and esophagus specimens obtained from BALB/c and C57BL/6 mice (Figures 3(a) and 3(b)).

Considering that nonclassical histocompatibility genes may be influenced by the action of inflammatory mediators, we evaluated the transcriptional levels of* INF-γ*,* NOS-2,* and* IL-10* genes in the heart to observe the relationship between these genes and the nonclassical genes.

*INF-γ*,* NOS-2,* and* IL-10* expression was increased in both BALB/c and C57BL/6 strains during the acute infection compared to controls and early chronically infected animals (*P* < 0.05; Figure 3(c)). The comparisons between strains showed that* INF-γ* expression was 4-fold higher in C57BL/6 than in BALB/c (Figure 3(c)). During the early chronic infection, the transcriptional levels of these genes were similar to noninfected mice for both BALB/c and C57BL/6 strains (Figure 3(c)). When we studied the relationship between these genes, we observed a positive correlation between* Qa2* and* INF-γ* (*P* = 0.0417; *r* = 1.0000) and* Qa2* and* NOS-2* (*P* = 0.0417; *r* = 1.0000) in heart specimens of both mouse strains during acute* T. cruzi* infection.

## 4. Discussion

The present study provides consistent data regarding the involvement of HLA-G and its Qa2 murine functional homolog in the* T. cruzi* infection.* HLA-G* gene polymorphism and HLA-G tissue expression have been evaluated in several diseases; however, little is known about the role of these molecules in the human or experimental* T. cruzi* infections.

The association of genetic markers with infectious disease has posed intriguing questions regarding the comparisons of gene frequencies between individuals with the disease with healthy individuals who had never been exposed to the specific infectious agent. Due to the myriad of chronic clinical forms observed in Chagas disease, part of these concerns may be circumvented by the possibility of comparing patients presenting well recognized clinical variants with patients who were infected by* T. cruzi* and have not developed clinically detected forms (indeterminate). When patients as a whole were compared to healthy individuals, some* HLA-G* 3′UTR alleles/genotypes were overrepresented (+3003T allele and +3003TT and +3187GG genotypes), while others were underrepresented (+3003C allele, and +3003CT, +3010GC, +3042GC genotypes) in Chagas disease patients. When symptomatic patients (CDM) were compared to healthy controls, similar results were obtained, indicating that these polymorphic sites were indeed associated with the clinically detected Chagas disease. In contrast, when indeterminate patients were compared to controls, the frequency of the* HLA-G* 3′UTR alleles/genotypes was closely similar. This result corroborates the role of the aforementioned* HLA-G* 3′UTR alleles and genotypes in the clinically recognized Chagas variants.

The stratification of Chagas patients into clinical variants showed that the +3027CC and +3035CC genotypes and the +3027C and +3035C alleles were strongly associated with the digestive form of Chagas disease. In addition, the +3142GC genotype was associated with a decreased risk to cardiac form development, when compared to the digestive form. These results indicate that genetic susceptibility to digestive or cardiac forms may be distinct. Indeed, previous studies conducted by our group evaluating classical histocompatibility (HLA-A, -B, -DRB1 and DQB1) antigens/alleles [55] and other immunoregulatory genes, such as* CTLA-4* [25], also showed a differential pattern of susceptibility according to the Chagas clinical variant. No specific posttranscription regulation mechanism has been reported concerning the +3027A/C and +3035C/T polymorphic sites; however, in a previous* in silico* study, we reported several miRNAs that may target the +3027A/C and +3035C/T polymorphic sites [56].

When* HLA-G* 3′UTR polymorphic sites were considered as haplotypes, decreased UTR-4 and UTR-7 frequencies were associated with the clinically detectable Chagas disease and with the digestive form, respectively. On the other hand, UTR-14 was associated with the cardiac variant and UTR-13 with the indeterminate form. Noteworthy, particular posttranscription elements that are present in these UTRs also exhibited a similar differential behavior when their frequencies were individually evaluated, further corroborating the role of* HLA-G* 3′UTR sites in disease susceptibility. Our group demonstrated that specific* HLA-G* 3′UTR haplotypes are associated with differential soluble levels of HLA-G (sHLA-G), being UTR-1 associated with high sHLA-G and UTR-5 and UTR-7 with decreased sHLA-G levels [40]. However, there are no studies associating* HLA-G* 3′UTR haplotypes with tissue HLA-G expression.

Considering that cytokines, chemokine, and immunomodulatory molecules play an important role in the pathogenesis of many diseases, including Chagas disease, and gene polymorphisms may influence the expression of these molecules, different studies have reported the association between different clinical forms of Chagas disease and polymorphisms at* TNF* [57–61],* IL-1β* [62],* IL-10* [63],* IL-4* [64],* INF-γ* [65],* TGF-β1* [66],* IL-12B* [67],* CXCL9* [68],* CXCL10* [68],* CCR5* [68–70],* CCL2* [71], and* CTLA-4* [25] genes.

Regarding HLA-G expression on tissue cells, patients with the digestive form exhibited decreased expression in colon but not in esophagus specimens, contrasting with patients exhibiting the cardiac variant who presented a decreased expression on myocardium cells. Many segments of the digestive tract and normal myocardium fibers [28] may constitutively express HLA-G; however, the meaning of these findings has not been completely elucidated. The best characterized constitutive HLA-G expression is in the placenta, where it may provide protection to the fetus against the cytotoxic cell immune response against paternal antigens [72]. HLA-G constitutive expression in gut and heart may in fact present a similar protective effect. Indeed, increased plasma levels of sHLA-G and myocardium HLA-G expression have been associated with decreased cellular acute rejection and better graft survival in patients submitted to heart transplantation [73–75]. Then, the loss of HLA-G expression on myocardium cells may facilitate the action of infiltrating lymphomononuclear cells after tissue injury caused by* T. cruzi*. On the other hand, myocardium infiltrating lymphomononuclear cells also exhibited HLA-G expression, which could decrease their functional properties. Since myocardium damage has been primarily attributed to fiber losses due to the action of immune cytotoxic cells, one may consider that HLA-G lymphomononuclear cell expression may not be the only regulatory surface cell molecule. In addition, it is important to emphasize that all these findings were observed in heart specimens of deceased patients, and the temporal HLA-G expression during the chronic phase has not been evaluated as yet.

Overall, the outcome of the experimental* T. cruzi* infection may depend on several factors, including (i) the inoculated strain, (ii) the amount of parasites, and (iii) the genetic background of the animal. Several of these combinations have been used to induce acute or early chronic infection. BALB/c mouse has been considered to be susceptible to acute* T. cruzi* infection induced by the Y and Tulahuén strains, due to the predominance of a Th2 immune response, characterized by high production of IL-4 and IL-10 [3, 76]. On the other hand, C57BL/6 mouse is considered to be resistant to acute* T. cruzi* infection induced by Y and Tulahuén strains, since the mouse produces a Th1 immune response with the production of INF-*γ*, IL-12, TNF, and NO [3, 14, 15]. In the present study, we used the less virulent* T. cruzi* CL strain that depending on the dose may produce severe acute or lead to the development of early chronic infection. In BALB/c mice, the acute infection by CL strain induced higher heart parasitism and parasitemia with increased production of proinflammatory mediators [77]. Since BALB/c is more susceptible to* T. cruzi* infection, chronic infection is difficult to be induced by more virulent strains. On the other hand, little is known about the acute and early chronic infection induced by CL strain in C57BL/6 mice. With the use of the CL strain we did not observe a differential susceptibility in BALB/c and C57BL/6 mice in terms of mouse survival; however, BALB/c showed higher parasitemia, whereas C57BL/6 presented higher heart parasitism. Therefore, the C57BL/6 controlled CL strain parasitemia but not heart tropism, and the inverse occurred with the BALB/c mouse.

Similarly to HLA-G, the Qa2 molecule may also control the immune response by inhibiting NK cells [45]. During acute* T. cruzi* infection, the transcriptional level of* Qa2* was increased in heart and esophagus in both BALB/c and C57BL/6 mouse strains, and the increased expression was not related to resistance or susceptibility to the acute infection. Although little attention has been devoted to the role of immunomodulatory nonclassical MHC molecules in experimental infection,* Qa2* expression may be induced to counterbalance the action of proinflammatory and other anti-inflammatory or immunomodulatory molecules. Indeed, the transcriptional levels of the* Qa1*,* CTLA-4*,* PDCD1*,* INF-γ*,* NOS-2,* and* IL-10* genes were also induced in the acute* T. cruzi* infection, as shown in this study. Additional literature findings corroborate this idea. Tissue damage observed in the affected tissues is due to the inflammatory process generated by an exacerbated immune response triggered against the parasite, and the high production of CTLA-4, PD-1, and IL-10 is related to control inflammatory process during* T. cruzi* infection [3, 14, 15, 23, 24, 78]. Our data suggest that the augmentation of immunomodulatory gene expression during the acute phase, including* Qa2* gene, may be associated with the control of tissue damage caused by inflammatory process, cytolysis, and fibrosis.

Regarding the early chronic infection, the expression of immunomodulatory, proinflammatory, and anti-inflammatory genes analyzed in this study was closely similar to noninfected mice in both BALB/c and C57BL/6 strains, and both animal groups were able to evolve into the early chronic infection, presenting only a little amount of* T. cruzi* in the heart. Considering that mice were exposed to decreased number of the less virulent CL strain, it is reasonable to conclude that both BALB/c and C57BL/6 mice had time and chance to mount an adequate immune response against the parasites.

## 5. Conclusion

We present several lines of evidence pointing to the participation of the immunomodulatory molecules HLA-G and its functional murine homolog Qa2 on human and experimental* T. cruzi* infection. In terms of Chagas disease susceptibility,* HLA-G* 3′UTR alleles/genotypes/haplotypes exhibited differential frequencies in infected/diseased patients when compared to only infected patients and healthy controls. The observation of a decreased HLA-G expression on cardiac muscle and colonic cells associated with the increased expression of HLA-G on myocardium infiltrating lymphomononuclear cells may reflect a lack of protection of these tissues that constitutively express HLA-G. In experimental infection, the mouse genetic background exerted only a mild influence on the overall response against the* T. cruzi* CL strain. The transcriptional levels of* Qa2*,* Qa1*,* CTLA-4*,* PDCD1*,* INF-γ*,* NOS-2,* and* IL-10* genes were induced only during the acute* T. cruzi* infection in BALB/c and C57BL/6 mice, indicating a fine balance between pro- and anti-inflammatory genes.

## Figures and Tables

**Table 1:** Genotype and allele frequencies of variation sites at *HLA-G* 3′ untranslated region (3′UTR) in patients with Chagas disease stratified according to clinical forms and healthy individuals.

3′UTR variation sites	Clinical forms													
	I		C		D		M		CDM		W		H	
	*n*	Freq.	*n*	Freq.	*n*	Freq.	*n*	Freq.	*n*	Freq.	*n*	Freq.	*n*	Freq.
14 bp I/D	(*n* = 39)		(*n* = 52)		(*n* = 62)		(*n* = 24)		(*n* = 138)		(*n* = 177)		(*n* = 155)	
II	8	0.2051	14	0.2692	8	0.1290	5	0.2083	27	0.1957	35	0.1977	30	0.1935
DI	19	0.4872	20	0.3846	32	0.5161	12	0.5000	64	0.4638	83	0.4689	67	0.4323
DD	12	0.3077	18	0.3462	22	0.3548	7	0.2917	47	0.3406	59	0.3333	58	0.3742
I allele	35	0.4487	48	0.4615	48	0.3871	22	0.4583	118	0.4275	153	0.4322	127	0.4097
D allele	43	0.5513	56	0.5385	76	0.6129	26	0.5417	158	0.5725	201	0.5678	183	0.5903

+3001C/T	(*n* = 39)		(*n* = 52)		(*n* = 62)		(*n* = 24)		(*n* = 138)		(*n* = 177)		(*n* = 155)	
CC	39	1.0000	52	1.0000	61	0.9839	24	1.0000	137	0.9928	176	0.9944	155	1.0000
CT	0	0.0000	0	0.0000	1	0.0161	0	0.0000	1	0.0072	1	0.0056	0	0.0000
TT	0	0.0000	0	0.0000	0	0.0000	0	0.0000	0	0.0000	0	0,0000	0	0.0000
C allele	78	1.0000	104	1.0000	123	0.9919	48	1.0000	275	0.9964	353	0.9972	310	1.0000
T allele	0	0.0000	0	0.0000	1	0.0081	0	0.0000	1	0.0036	1	0.0028	0	0.0000

+3003T/C	(*n* = 39)		(*n* = 52)		(*n* = 62)		(*n* = 24)		(*n* = 138)		(*n* = 177)		(*n* = 155)	
TT	32	0.8205	45	0.8654	54	0.8710^*^	20	0.8333	119	0.8623^*^	151	0.8531^*^	114	0.7354^*^
CT	7	0.1795	7	0.1346	8	0.1290^*^	4	0.1667	19	0.1377^*^	26	0.1469^*^	40	0.2581^*^
CC	0	0.0000	0	0.0000	0	0.0000	0	0.0000	0	0.0000	0	0.0000	1	0.0065
T allele	71	0.9103	97	0.9327	116	0.9355^*^	44	0.9167	257	0.9312^*^	328	0.9266^*^	268	0.8645^*^
C allele	7	0.0897	7	0.0673	8	0.0645^*^	4	0.0833	19	0.0688^*^	26	0.0734^*^	42	0.1355^*^

+3010C/G	(*n* = 39)		(*n* = 52)		(*n* = 62)		(*n* = 24)		(*n* = 138)		(*n* = 177)		(*n* = 155)	
CC	15	0.3846	21	0.4038	16	0.2581	10	0.4167	47	0.3406	62	0.3503	44	0.2839
GC	14	0.3590	16	0.3077^*^	31	0.5000	9	0.3750	56	0.4058	70	0.3955^*^	74	0.4774^*^
GG	10	0.2564	15	0.2885	15	0.2419	5	0.2083	35	0.2536	45	0.2542	37	0.2387
C allele	44	0.5641	58	0.5577	63	0.5081	29	0.6042	150	0.5435	194	0.5480	162	0.5226
G allele	34	0.4359	46	0.4423	61	0.4919	19	0.3958	126	0.4565	160	0.4520	148	0.4774

+3027A/C	(*n* = 39)		(*n* = 52)		(*n* = 62)		(*n* = 24)		(*n* = 138)		(*n* = 177)		(*n* = 155)	
AA	0	0.0000	0	0.0000	0	0.0000	0	0.0000	0	0.0000	0	0.0000	1	0.0065
AC	6	0.1538^*^	6	0.1154^*^	0	0.0000^*^	4	0.1667^*^	10	0.0725	16	0.0904	15	0.0968^*^
CC	33	0.8462	46	0.8846	62	1.0000	20	0.8333	128	0.9275	161	0.9096	139	0.8967^*^
A allele	6	0.0769^*^	6	0.0577^*^	0	0.0000^*^	4	0.0833^*^	10	0.0362	16	0.0452	17	0.0548^*^
C allele	72	0.9231^*^	98	0.9423^*^	124	1.0000^*^	44	0.9167^*^	266	0.9638	338	0.9548	293	0.9452^*^

+3035C/T	(*n* = 39)		(*n* = 52)		(*n* = 62)		(*n* = 24)		(*n* = 138)		(*n* = 177)		(*n* = 155)	
CC	30	0.7692	38	0.7308^*^	56	0.9032^*^	17	0.7083^*^	111	0.8043	141	0.7966	111	0.7161^*^
CT	7	0.1795	12	0.2308	6	0.0968^*^	7	0.2917^*^	25	0.1812	32	0.1808	40	0.2581^*^
TT	2	0.0513	2	0.0384	0	0.0000	0	0.0000	2	0.0145	4	0.0226	4	0.0258
C allele	67	0.8590^*^	88	0.8462^*^	118	0.9516^*^	41	0.8542^*^	247	0.8949	314	0.8870	262	0.8452^*^
T allele	11	0.1410^*^	16	0.1538^*^	6	0.0484^*^	7	0.1458^*^	29	0.1051	40	0.1130	48	0.1548^*^

+3142G/C	(*n* = 39)		(*n* = 51)		(*n* = 60)		(*n* = 24)		(*n* = 135)		(*n* = 174)		(*n* = 155)	
GG	16	0.4103^*^	21	0.4118^*^	13	0.2166^*^	11	0.4583^*^	45	0.3333	61	0.3505	44	0.2839
GC	15	0.3846	15	0.2941^*^	31	0.5167^*^	8	0.3333	54	0.4000	69	0.3966^*^	80	0.5161^*^
CC	8	0.2051	15	0.2941	16	0.2667	5	0.2084	36	0.2667	44	0.2529	31	0.2000
G allele	47	0.6026	57	0.5588	57	0.4750	30	0.6250	144	0.5333	191	0.5489	168	0.5419
C allele	31	0.3974	45	0.4412	63	0.5250	18	0.3750	126	0.4667	157	0.4511	142	0.4581

+3187A/G	(*n* = 39)		(*n* = 50)		(*n* = 61)		(*n* = 24)		(*n* = 135)		(*n* = 174)		(*n* = 155)	
AA	22	0.5641	27	0.5400	26	0.4262	14	0.5833	67	0.4963	89	0.5115	82	0.5290
GA	12	0.3077	16	0.3200	29	0.4754	7	0.2917	52	0.3852	64	0.3678	66	0.4258
GG	5	0.1282	7	0.1400^*^	6	0.0984	3	0.1250	16	0.1185^*^	21	0.1207^*^	7	0.0452^*^
A allele	56	0.7179	70	0.7000	81	0.6639	35	0.7292	186	0.6889	242	0.6954	230	0.7419
G allele	22	0.2821	30	0.3000	41	0.3361	13	0.2708	84	0.3111	106	0.3046	80	0.2581

+3196C/G	(*n* = 39)		(*n* = 50)		(*n* = 61)		(*n* = 24)		(*n* = 135)		(*n* = 174)		(*n* = 155)	
CC	22	0.5641	24	0.4800	25	0.4098	8	0.3333^*^	57	0.4222^*^	79	0.4540	86	0.5548^*^
GC	12	0.3077^*^	22	0.4400	31	0.5082	15	0.6250^*^	68	0.5037^*^	80	0.4598	58	0.3742^*^
GG	5	0.1282	4	0.0800	5	0.0820	1	0.0417	10	0.0741	15	0.0862	11	0.0710
C allele	56	0.7179	70	0.7000	81	0.6639	31	0.6458	182	0.6741	238	0.6839	230	0.7419
G allele	22	0.2821	30	0.3000	41	0.3361	17	0.3542	88	0.3259	110	0.3161	80	0.2581

I (indeterminate). C (cardiac). D (digestive). M (mixed). CDM (patients presenting clinically detected disease). W (whole group). H (healthy control). ^*^frequencies that show statistical differences.

**Table 2:** Odds Ratio and 95% Confidence Interval values obtained from the comparisons of genotype and allele frequencies of variation sites at *HLA*-*G* 3′ untranslated region (3′ UTR) between different presentation forms of Chagas disease and healthy controls.

Genotypes and alleles	Comparison	OR (95% CI)	Comparison	OR (95% CI)	Comparison	OR (95% CI)
+3003CT	W versus H	0.495 (0.29–0.86)	CDM versus H	0.459 (0.25–0.84)	D versus H	0.426 (0.19–0.97)
+3003TT	W versus H	2.089 (1.21–3.61)	CDM versus H	2.252 (1.23–4.11)	D versus H	2.428 (1.07–5.53)
+3003C	W versus H	0.506 (0.30–0.85)	CDM versus H	0.472 (0.27–0.83)	D versus H	0.440 (0.20–0.97)
+3003T	W versus H	1.977 (1.18–3.31)	CDM versus H	2.119 (1.20–3.74)	D versus H	2.272 (1.03–4.99)
+3010GC	W versus H	0.610 (0.39–0.95)	C versus H	0.414 (0.21–0.81)		
+3027AC	D versus I	0.041 (0.002–0.75)	D versus M	0.036 (0.002–0.71)	C versus D	17.473 (0.96–318.00)
	D versus H	0.073 (0.004–1.23)				
+3027CC	D versus I	24.254 (1.33–443.84)	D versus M	27.439 (1.42–531.65)	C versus D	0.057 (0.003–1.04)
	D versus H	14.785 (0.87–250.35)				
+3027A	D versus I	0.045 (0.002–0.81)	D versus M	0.040 (0.002–0.75)	C versus D	16.431 (0.91–295.23)
	D versus H	0.067 (0.004–1.13)				
+3027C	D versus I	22.324 (1.24–402.08)	D versus M	25.180 (1.33–477.12)	C versus D	0.061 (0.003–1.09)
	D versus H	14.847 (0.89–248.81)				
+3035CC	D versus M	3.843 (1.14–12.99)	C versus D	0.291 (0.10–0.82)	D versus H	3.700 (1.49–9.20)
+3035CT	D versus M	0.260 (0.08–0.88)	D versus H	0.308 (0.12–0.77)		
+3035C	D versus I	3.229 (1.14–9.13)	D versus M	3.358 (1.07–10.57)	C versus D	0.280 (0.11–0.74)
	D versus H	3.603 (1.50–8.65)				
+3035T	D versus I	0.310 (0.11–0.88)	D versus M	0.298 (0.09–0.94)	C versus D	3.576 (1.34–9.51)
	D versus H	0.278 (0.12–0.67)				
+3142GC	W versus H	0.616 (0.40–0.95)	C versus D	0.390 (0.18–0.86)	C versus H	0.391 (0.20–0.77)
+3142GG	D versus I	0.398 (0.16–0.96)	D versus M	0.327 (0.12–0.90)	C versus D	2.531 (1.10–5.80)
+3187GG	W versus H	2.902 (1.20–7.03)	CDM versus H	2.843 (1.13–7.14)	C versus H	3.442 (1.14–10.35)
+3196CC	CDM versus H	0.586 (0.37–0.93)	M versus H	0.401 (0.16–0.99)		
+3196GC	CDM versus H	1.697 (1.06–2.71)	CDM versus I	2.284 (1.07–4.88)	I versus M	0.267 (0.09–0.78)
	M versus H	2.787 (1.15–6.77)				

I (indeterminate). C (cardiac). D (digestive). M (mixed). CDM (patients presenting clinically detected disease). W (whole group). H (healthy control). OR, Odds Ratio. 95% CI, 95% Confidence Interval.

**(a):** 

Haplotype		Clinical forms													
		I		C		D		M		CDM		W		H	
		*n *	Freq.	*n *	Freq.	*n *	Freq.	*n *	Freq.	*n *	Freq.	*n *	Freq.	*n *	Freq.
UTR-1	DelCTGCCCGC	17	0.25	27	0.28	40	0.32	80	0.26	80	0.30	80	0.26	80	0.26
UTR-2	InsCTCCCGAG	20	0.29	28	0.29	37	0.31	75	0.24	80	0.30	75	0.24	75	0.24
UTR-3	DelCTCCCGAC	11	0.16	12	0.12	13	0.11	39	0.13	32	0.12	39	0.13	39	0.13
UTR-4	DelCCGCCCAC	4	0.06	3	0.03^*^	7	0.06^*^	41	0.13	13	0.05^*^	41	0.13^*^	41	0.13^*^
UTR-5	InsCTCCTGAC	3	0.04	11	0.11	5	0.04	29	0.09	18	0.07	29	0.09	29	0.09
UTR-6	DelCTGCCCAC	3	0.04	6	0.06	11	0.09	21	0.07	19	0.07	21	0.07	21	0.07
UTR-7	InsCTCATGAC	5	0.07^*^	3	0.03	0	0.00^*^	17	0.05^*^	7	0.03	17	0.05	17	0.05^*^
UTR-8	InsCTGCCGAG	1	0.02	0	0.00	0	0.00	4	0.01	0	0.00	4	0.01	4	0.01
UTR-10	DelCTCCCGAG	0	0.00	0	0.00	1	0.01	1	0.003	1	0.004	1	0.003	1	0.003
UTR-11	DelCCCCCGAC	0	0.00	0	0.00	0	0.00	1	0.003	0	0.00	1	0.003	1	0.003
UTR-13	DelCTCCTGAC	2	0.03^*^	0	0.00	0	0.00	0	0.00	0	0.00^*^	0	0.00	0	0.00^*^
UTR-14	DelCTGCCGGC	1	0.02	3	0.03^*^	0	0.00	0	0.00	3	0.01	0	0.00	0	0.00^*^
UTR-17	InsTTCCTGAC	0	0.00	0	0.00	1	0.01	0	0.00	1	0.004	0	0.00	0	0.00
Others^a^	DelCCGCCCGC	0	0.00	1	0.01	0	0.00	0	0.00	1	0.004	0	0.00	0	0.00
	DelCTCACCAC	0	0.00	1	0.01	0	0.00	0	0.00	1	0.004	0	0.00	0	0.00
	DelCTCCCCAC	0	0.00	0	0.00	1	0.01	0	0.00	1	0.004	0	0.00	0	0.00
	DelCTCCCCGC	0	0.00	0	0.00	1	0.01	0	0.00	1	0.004	0	0.00	0	0.00
	DelCCGACCGC	0	0.00	1	0.01	0	0.00	0	0.00	1	0.004	0	0.00	0	0.00
	InsCTCCCCAG	0	0.00	0	0.00	2	0.02	0	0.00	2	0.01	0	0.00	0	0.00
	InsCTGCCCAC	0	0.00	2	0.02	0	0.00	0	0.00	2	0.01	0	0.00	0	0.00
	InsCTGCCCGC	1	0.02	0	0.00	1	0.01	0	0.00	1	0.004	0	0.00	0	0.00

**(b):** 

Haplotype	Comparison	OR (95% CI)	Comparison	OR (95% CI)	Comparison	OR (95% CI)
UTR-4	W versus H	0.354 (0.20–0.64)	CDM versus H	0.339 (0.18–0.65)	D versus H	0.406 (0.18–0.93)
	C versus H	0.207 (0.06–0.69)				
UTR-7	D versus I	0.048 (0.003–0.88)	D versus M	0.039 (0.002–0.74)	D versus H	0.070 (0.004–1.17)
UTR-13	CDM versus I	0.050 (0.002–1.06)	I versus H	23.346 (1.08–491.92)		
UTR-14	C versus H	22.759 (1.17–444.53)				

I (indeterminate). C (cardiac). D (digestive). M (mixed). CDM (patients presenting clinically detected disease). W (whole group). H (healthy control). Del (deletion), Ins (insertion), OR, Odds Ratio, 95% CI, 95% Confidence Interval. ^*^frequencies that show statistical differences. ^a^Group of haplotypes occurring at a frequency of ≤0.02.

## References

[1] Marin-Neto J. A., Rassi A. (2009). Update on chagas heart disease on the first centennial of its discovery. *Revista Española de Cardiología*.

[2] Boscardin S. B., Torrecilhas A. C. T., Manarin R. (2010). Chagas' disease: an update on immune mechanisms and therapeutic strategies. *Journal of Cellular and Molecular Medicine*.

[3] Rassi A., Rassi A., Marin-Neto J. A. (2010). Chagas disease. *The Lancet*.

[4] Ribeiro B. M., Crema E., Rodrigues V. (2008). Analysis of the cellular immune response in patients with the digestive and indeterminate forms of Chagas' disease. *Human Immunology*.

[5] Leavey J. K., Tarleton R. L. (2003). Cutting edge: Dysfunctional CD8+ T cells reside in nonlymphoid tissues during chronic *Trypanosoma cruzi* infection. *Journal of Immunology*.

[6] Lannes-Vieira J. (2003). Trypanosoma cruzi-elicited CD8^+^ T Cell-mediated Myocarditis: chemokine receptors and adhesion molecules as potential therapeutic targets to control chronic inflammation?. *Memorias do Instituto Oswaldo Cruz*.

[7] Bryan M. A., Guyach S. E., Norris K. A. (2010). Specific humoral immunity versus polyclonal B Cell activation in *Trypanosoma cruzi* infection of susceptible and resistant mice. *PLoS Neglected Tropical Diseases*.

[8] Benítez-Hernández I., Méndez-Enríquez E., Ostoa P. (2010). Proteolytic cleavage of chemokines by *Trypanosoma cruzi's* cruzipain inhibits chemokine functions by promoting the generation of antagonists. *Immunobiology*.

[9] Gorelik G., Cremashi G., Borda E., Sterin-Borda L. (1998). Trypanosoma cruzi antigens down-regulate T lymphocyte proliferation by muscarinic cholinergic receptor-dependent release of PGE2. *Acta Physiologica Pharmacologica et Therapeutica Latinoamericana*.

[10] Borges M., Da Silva A. C., Sereno D., Ouaissi A. (2003). Peptide-based analysis of the amino acid sequence important to the immunoregulatory function of *Trypanosoma cruzi* Tc52 virulence factor. *Immunology*.

[11] Cunha-Neto E., Coelho V., Guilherme L., Fiorelli A., Stolf N., Kalil J. (1996). Autoimmunity in Chagas' disease: identification of cardiac myosin-B13 Trypanosoma cruzi protein crossreactive T cell clones in heart lesions of a chronic Chagas' cardiomyopathy patient. *Journal of Clinical Investigation*.

[12] Khoury E. L., Ritacco V., Cossio P. M. (1979). Circulating antibodies to peripheral nerve in American trypanosomiasis (Chagas' disease). *Clinical and Experimental Immunology*.

[13] Kurup S. P., Tarleton R. L. (2013). Perpetual expression of PAMPs necessary for optimal immune control and clearance of a persistent pathogen. *Nature Communications*.

[14] Brener Z., Gazzinelli R. T. (1997). Immunological control of *Trypanosoma cruzi* infection and pathogenesis of Chagas' disease. *International Archives of Allergy and Immunology*.

[15] Gutierrez F. R. S., Mineo T. W. P., Pavanelli W. R., Guedes P. M. M., Silva J. S. (2009). The effects of nitric oxide on the immune system during Trypanosoma cruzi infection. *Memorias do Instituto Oswaldo Cruz*.

[16] Abel L. C. J., Rizzo L. V., Ianni B. (2001). Chronic Chagas' disease cardiomyopathy patients display an increased IFN-*γ* response to *Trypanosoma cruzi* infection. *Journal of Autoimmunity*.

[17] Gomes J. A. S., Bahia-Oliveira L. M. G., Rocha M. O. C., Martins-Filho O. A., Gazzinelli G., Correa-Oliveira R. (2003). Evidence that development of severe cardiomyopathy in human Chagas' disease is due to a Th1-specific immune response. *Infection and Immunity*.

[18] Teixeira M. M., Gazzinelli R. T., Silva J. S. (2002). Chemokines, inflammation and *Trypanosoma cruzi* infection. *Trends in Parasitology*.

[19] Talvani A., Rocha M. O. C., Barcelos L. S., Gomes Y. M., Ribeiro A. L., Teixeira M. M. (2004). Elevated concentrations of CCL2 and tumor necrosis factor-*α* in chagasic cardiomyopathy. *Clinical Infectious Diseases*.

[20] Dutra W. O., Menezes C. A., Magalhaes L. M., Gollob K. J. (2014). Immunoregulatory networks in human Chagas disease. *Parasite Immunology*.

[21] Martins G. A., Tadokoro C. E., Silva R. B., Silva J. S., Rizzo L. V. (2004). CTLA-4 blockage increases resistance to infection with the intracellular protozoan *Trypanosoma cruzi*. *Journal of Immunology*.

[22] Graefe S. E. B., Jacobs T., Wächter U., Bröker B. M., Fleischer B. (2004). CTLA-4 regulates the murine immune response to Trypanosoma cruzi infection. *Parasite Immunology*.

[23] Souza P. E. A., Rocha M. O. C., Menezes C. A. S. (2007). Trypanosoma cruzi infection induces differential modulation of costimulatory molecules and cytokines by monocytes and T cells from patients with indeterminate and cardiac Chagas' disease. *Infection and Immunity*.

[24] Gutierrez F. R., Mariano F. S., Oliveira C. J. F. (2011). Regulation of *Trypanosoma cruzi*-induced myocarditis by programmed death cell receptor 1. *Infection and Immunity*.

[25] Dias F. C., Medina T. D. S., Mendes-Junior C. T. (2013). Polymorphic sites at the immunoregulatory CTLA-4 gene are associated with chronic chagas disease and its clinical manifestations. *PLoS ONE*.

[26] González A., Rebmann V., Lemaoult J., Horn P. A., Carosella E. D., Alegre E. (2012). The immunosuppressive molecule HLA-G and its clinical implications. *Critical Reviews in Clinical Laboratory Sciences*.

[27] Rajagopalan S., Bryceson Y. T., Kuppusamy S. P. (2006). Activation of NK cells by an endocytosed receptor for soluble HLA-G. *PLoS Biology*.

[28] Onno M., Guillaudeux T., Amiot L. (1994). The HLA-G gene is expressed at a low mRNA level in different human cells and tissues. *Human Immunology*.

[29] McMaster M. T., Librach C. L., Zhou Y. (1995). Human placental HLA-G expression is restricted to differentiated cytotrophoblasts. *Journal of Immunology*.

[30] Mallet V., Fournel S., Schmitt C., Campan A., Lenfant F., Le Bouteiller P. (1999). Primary cultured human thymic epithelial cells express both membrane-bound and soluble HLA-G translated products. *Journal of Reproductive Immunology*.

[31] Le Discorde M., Moreau P., Sabatier P., Legeais J.-M., Carosella E. D. (2003). Expression of HLA-G in human cornea, an immune-privileged tissue. *Human Immunology*.

[32] Cirulli V., Zalatan J., McMaster M. (2006). The class I HLA repertoire of pancreatic islets comprises the nonclassical class Ib antigen HLA-G. *Diabetes*.

[33] Huang Y. H., Airas L., Schwab N., Wiendl H. (2011). Janus head: the dual role of HLA-G in CNS immunity. *Cellular and Molecular Life Sciences*.

[34] Menier C., Rabreau M., Challier J.-C., Le Discorde M., Carosella E. D., Rouas-Freiss N. (2004). Erythroblasts secrete the nonclassical HLA-G molecule from primitive to definitive hematopoiesis. *Blood*.

[35] Yan W.-H. (2011). Human leukocyte antigen-G in cancer: are they clinically relevant?. *Cancer Letters*.

[36] Tripathi P., Agrawal S. (2007). The role of human leukocyte antigen E and G in HIV infection. *AIDS*.

[37] Castelli E. C., Mendes-Junior C. T., Veiga-Castelli L. C., Roger M., Moreau P., Donadi E. A. (2011). A comprehensive study of polymorphic sites along the HLA-G gene: implication for gene regulation and evolution. *Molecular Biology and Evolution*.

[38] Sabbagh A., Luisi P., Castelli E. C. (2014). Worldwide genetic variation at the 3′ untranslated region of the HLA-G gene: balancing selection influencing genetic diversity. *Genes and Immunity*.

[39] Castelli E. C., Mendes-Junior C. T., Deghaide N. H. S. (2010). The genetic structure of 3′untranslated region of the HLA-G gene: polymorphisms and haplotypes. *Genes and Immunity*.

[40] Martelli-Palomino G., Pancotto J. A., Muniz Y. C. (2013). Polymorphic sites at the 3′ untranslated region of the HLA-G gene are associated with differential hla -g soluble levels in the Brazilian and French population. *PLoS ONE*.

[41] Flaherty L., Elliott E., Tine J. A., Walsh A. C., Waters J. B. (1990). Immunogenetics of the Q and TL regions of the mouse. *Critical Reviews in Immunology*.

[42] Melo-Lima B. L., Evangelista A. F., De Magalhães D. A. R., Passos G. A., Moreau P., Donadi E. A. (2014). Differential transcript profiles of MHC class Ib(Qa-1, Qa-2, and Qa-10) and aire genes during the ontogeny of thymus and other tissues. *Journal of Immunology Research*.

[43] Joyce S., Tabaczewski P., Angeletti R. H., Nathenson S. G., Stroynowski I. (1994). A nonpolymorphic major histocompatibility complex class Ib molecule binds a large array of diverse self-peptides. *Journal of Experimental Medicine*.

[44] Kumánovics A., Madan A., Qin S., Rowen L., Hood L., Lindahl K. F. (2002). Quod erat faciendum: sequence analysis of the H2-D and H2-Q regions of 129/SvJ mice. *Immunogenetics*.

[45] Chiang E. Y., Henson M., Stroynowski I. (2002). The nonclassical major histocompatibility complex molecule Qa-2 protects tumor cells from NK cell- and lymphokine-activated killer cell-mediated cytolysis. *The Journal of Immunology*.

[46] Silva T. G., Crispim J. C. O., Miranda F. A. (2011). Expression of the nonclassical HLA-G and HLA-E molecules in laryngeal lesions as biomarkers of tumor invasiveness. *Histology and Histopathology*.

[47] Tristão F. S. M., Rocha F. A., Moreira A. P., Cunha F. Q., Rossi M. A., Silvaa J. S. (2013). 5-Lipoxygenase activity increases susceptibility to experimental *Paracoccidioides brasiliensis* infection. *Infection and Immunity*.

[48] Cummings K. L., Tarleton R. L. (2003). Rapid quantitation of *Trypanosoma cruzi* in host tissue by real-time PCR. *Molecular and Biochemical Parasitology*.

[49] Bustin S. A., Benes V., Garson J. A. (2009). The MIQE guidelines: minimum information for publication of quantitative real-time PCR experiments. *Clinical Chemistry*.

[50] Raymond M., Rousset F. (1995). Genepop (version 1.2): population-genetics software for exact tests and ecumenicism. *Journal of Heredity*.

[51] Excoffier L., Lischer H. E. L. (2010). Arlequin suite ver 3.5: a new series of programs to perform population genetics analyses under Linux and Windows. *Molecular Ecology Resources*.

[52] Excoffier L., Slatkin M. (1995). Maximum-likelihood estimation of molecular haplotype frequencies in a diploid population. *Molecular Biology and Evolution*.

[53] Qin Z. S., Niu T., Liu J. S. (2002). Partition-ligation-expectation-maximization algorithm for haplotype inference with single-nucleotide polymorphisms. *The American Journal of Human Genetics*.

[54] Stephens M., Smith N. J., Donnelly P. (2001). A new statistical method for haplotype reconstruction from population data. *The American Journal of Human Genetics*.

[55] Deghaide N. H. S., Dantas R. O., Donadi E. A. (1998). HLA class I and II profiles of patients presenting with Chagas' disease. *Digestive Diseases and Sciences*.

[56] Castelli E. C., Moreau P., Chiromatzo A. O. E. (2009). In silico analysis of microRNAS targeting the HLA-G 3′ untranslated region alleles and haplotypes. *Human Immunology*.

[57] Rodríguez-Pérez J. M., Cruz-Robles D., Hernández-Pacheco G. (2005). Tumor necrosis factor-alpha promoter polymorphism in Mexican patients with Chagas' disease. *Immunology Letters*.

[58] Drigo S. A., Cunha-Neto E., Ianni B. (2006). TNF gene polymorphisms are associated with reduced survival in severe Chagas' disease cardiomyopathy patients. *Microbes and Infection*.

[59] Campelo V., Dantas R. O., Simões R. T. (2007). TNF microsatellite alleles in Brazilian chagasic patients. *Digestive Diseases and Sciences*.

[60] Pissetti C. W., Correia D., de Oliveira R. F. (2011). Genetic and functional role of TNF-alpha in the development *Trypanosoma cruzi* infection. *PLoS Neglected Tropical Diseases*.

[61] Criado L., Flórez O., Martín J., González C. I. (2012). Genetic polymorphisms in TNFA/TNFR2 genes and Chagas disease in a Colombian endemic population. *Cytokine*.

[62] Flórez O., Zafra G., Morillo C., Martín J., González C. I. (2006). Interleukin-1 gene cluster polymorphism in chagas disease in a Colombian case-control study. *Human Immunology*.

[63] Costa G. C., Rocha M. O. D. C., Moreira P. R. (2009). Functional IL-10 gene polymorphism is associated with Chagas disease cardiomyopathy. *Journal of Infectious Diseases*.

[64] Alvarado Arnez L. E., Venegas E. N., Ober C., Thompson E. E. (2011). Sequence variation in the *IL*
_4_ gene and resistance to *Trypanosoma cruzi* infection in Bolivians. *Journal of Allergy and Clinical Immunology*.

[65] Torres O. A., Calzada J. E., Beraún Y. (2010). Role of the IFNG +874T/A polymorphism in Chagas disease in a Colombian population. *Infection, Genetics and Evolution*.

[66] Calzada J. E., Beraún Y., González C. I., Martín J. (2009). Transforming growth factor beta 1 (TGF*β*1) gene polymorphisms and Chagas disease susceptibility in Peruvian and Colombian patients. *Cytokine*.

[67] Zafra G., Morillo C., Martín J., González A., González C. I. (2007). Polymorphism in the 3′ UTR of the IL12B gene is associated with Chagas' disease cardiomyopathy. *Microbes and Infection*.

[68] Nogueira L. G., Santos R. H. B., Ianni B. M. (2012). Myocardial chemokine expression and intensity of myocarditis in Chagas cardiomyopathy are controlled by polymorphisms in CXCL9 and CXCL10. *PLoS Neglected Tropical Diseases*.

[69] Calzada J. E., Nieto A., Beraún Y., Martín J. (2001). Chemokine receptor CCR5 polymorphisms and Chagas' disease cardiomyopathy. *Tissue Antigens*.

[70] Flórez O., Martín J., González C. I. (2012). Genetic variants in the chemokines and chemokine receptors in Chagas disease. *Human Immunology*.

[71] Ramasawmy R., Cunha-Neto E., Faé K. C. (2006). The monocyte chemoattractant protein-1 gene polymorphism is associated with cardiomyopathy in human Chagas disease. *Clinical Infectious Diseases*.

[72] Rouas-Freiss N., Kirszenbaum M., Dausset J., Carosella E. D. (1997). Fetal-maternal tolerance: Role of HLA-G in protection of the fetus against maternal natural killer cell activity. *Comptes Rendus de l'Academie des Sciences III*.

[73] Sheshgiri R., Rouas-Freiss N., Rao V. (2008). Myocardial HLA-G reliably indicates a low risk of acute cellular rejection in heart transplant recipients. *Journal of Heart and Lung Transplantation*.

[74] Lila N., Carpentier A., Amrein C., Khalil-Daher I., Dausset J., Carosella E. D. (2000). Implication of HLA-G molecule in heart-graft acceptance. *The Lancet*.

[75] Lila N., Amrein C., Guillemain R. (2002). Human leukocyte antigen-G expression after heart transplantation is associated with a reduced incidence of rejection. *Circulation*.

[76] Hoft D. F., Lynch R. G., Kirchhoff L. V. (1993). Kinetic analysis of antigen-specific immune responses in resistant and susceptible mice during infection with *Trypanosoma cruzi*. *Journal of Immunology*.

[77] Rodrigues C. M., Valadares H. M. S., Francisco A. F. (2010). Coinfection with different Trypanosoma cruzi strains interferes with the host immune response to infection. *PLoS Neglected Tropical Diseases*.

[78] de Araújo F. F., Vitelli-Avelar D. M., Teixeira-Carvalho A. (2011). Regulatory T cells phenotype in different clinical forms of chagas' disease. *PLoS Neglected Tropical Diseases*.

